# Supplementary material for: Surface Microbiome Profiling of Dental Elevators Using Third‐Generation Sequencing: Implications for Infection Control in Dental Practice
Source: Microbiologyopen. 2025 Dec 8;14(6):e70178. doi: 10.1002/mbo3.70178 (PMC12685768; doi:10.1002/mbo3.70178)
Supplement: Supplementary file 1 — customer_backup. [file MBO3-14-e70178-s001.zip › customer_backup/customer_backup/function_analysis/COG/allsample/K2species/allsample.reabundance_Class2Species.txt.pdf]

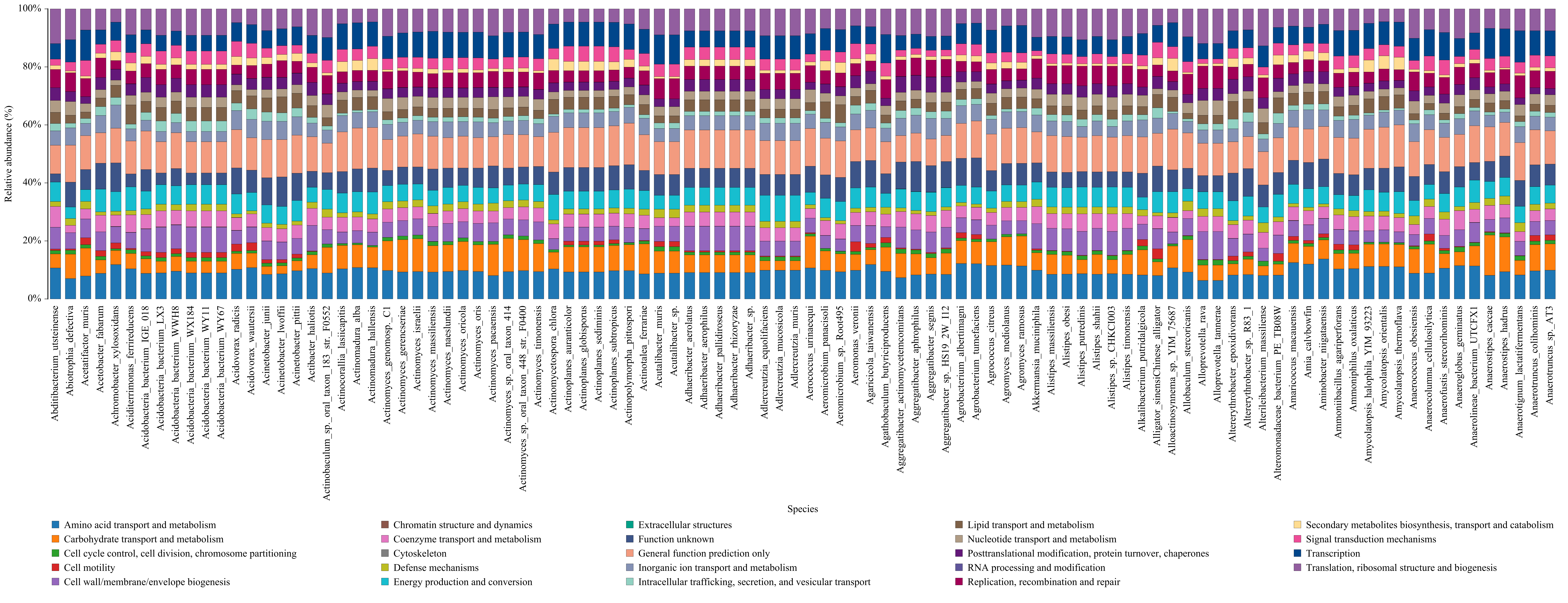
100%
80%
60%
40%
20%
0%
Relative abundance (%)
Abditibacterium_utsteinense
Abiotrophia_defectiva
Acetatifactor_muris
Acetobacter_fabarum
Achromobacter_xylosoxidans
Acidiferrimonas_ferrireducens
Acidobacteria_bacterium_IGE_018
Acidobacteria_bacterium_LX3
Acidobacteria_bacterium_WWH8
Acidobacteria_bacterium_WX184
Acidobacteria_bacterium_WY11
Acidobacteria_bacterium_WY67
Acidovorax_radicis
Acidovorax_wautersii
Acinetobacter_junii
Acinetobacter_lwoffii
Acinetobacter_pittii
Actibacter_haliotis
Actinobaculum_sp._oral_taxon_183_str._F0552
Actinocorallia_lasiicapitis
Actinomadura_alba
Actinomadura_hallensis
Actinomyces_genomosp._C1
Actinomyces_gerencseriae
Actinomyces_israelii
Actinomyces_massiliensis
Actinomyces_naeslundii
Actinomyces_oricola
Actinomyces_oris
Actinomyces_pacaensis
Actinomyces_sp._oral_taxon_414
Actinomyces_sp._oral_taxon_448_str._F0400
Actinomyces_timonensis
Actinomycetospora_chlora
Actinoplanes_auranticolor
Actinoplanes_globisporus
Actinoplanes_sediminis
Actinoplanes_subtropicus
Actinopolymorpha_pittospori
Actinotalea_ferrariae
Acutalibacter_muris
Acutalibacter_sp.
Adhaeribacter_aerolatus
Adhaeribacter_aerophilus
Adhaeribacter_pallidiroseus
Adhaeribacter_rhizoryzae
Adhaeribacter_sp.
Adlercreutzia_equolifaciens
Adlercreutzia_mucosicola
Adlercreutzia_muris
Aerococcus_urinaeequi
Aeromicrobium_panaciisoli
Aeromicrobium_sp._Root495
Aeromonas_veronii
Agaricicola_taiwanensis
Agathobaculum_butyriciproducens
Aggregatibacter_actinomycetemcomitans
Aggregatibacter_aphrophilus
Aggregatibacter_segnis
Aggregatibacter_sp._HS19_2W_I12
Agrobacterium_albertimagni
Agrobacterium_tumefaciens
Agrococcus_citreus
Agromyces_mediolanus
Agromyces_ramosus
Akkermansia_muciniphila
Alistipes_massiliensis
Alistipes_obesi
Alistipes_putredinis
Alistipes_shahii
Alistipes_sp._CHKCI003
Alistipes_timonensis
Alkalibacterium_putridalgicola
Alligator_sinensiChinese_alligator
Alloactinosynnema_sp._YIM_75687
Allobaculum_stercoricanis
Alloprevotella_rava
Alloprevotella_tannerae
Altererythrobacter_epoxidivorans
Altererythrobacter_sp._R83_1
Alterileibacterium_massiliense
Alteromonadaceae_bacterium_PE_TB08W
Amaricoccus_macauensis
Amia_calvbowfin
Aminobacter_niigataensis
Ammonibacillus_agariperforans
Ammoniphilus_oxalaticus
Amycolatopsis_halophila_YIM_93223
Amycolatopsis_orientalis
Amycolatopsis_thermoflava
Anaerococcus_obesiensis
Anaerocolumna_cellulosilytica
Anaerofustis_stercorihominis
Anaeroglobus_geminatus
Anaerolineae_bacterium_UTCFX1
Anaerostipes_caccae
Anaerostipes_hadrus
Anaerotignum_lactatifermentans
Anaerotruncus_colihominis
Anaerotruncus_sp._AT3
Species
Amino acid transport and metabolism
Carbohydrate transport and metabolism
Cell cycle control, cell division, chromosome partitioning
Cell motility
Cell wall/membrane/envelope biogenesis
Chromatin structure and dynamics
Coenzyme transport and metabolism
Cytoskeleton
Defense mechanisms
Energy production and conversion
Extracellular structures
Function unknown
General function prediction only
Inorganic ion transport and metabolism
Intracellular trafficking, secretion, and vesicular transport
Lipid transport and metabolism
Nucleotide transport and metabolism
Posttranslational modification, protein turnover, chaperones
RNA processing and modification
Replication, recombination and repair
Secondary metabolites biosynthesis, transport and catabolism
Signal transduction mechanisms
Transcription
Translation, ribosomal structure and biogenesis
